# Increased Total Serum Immunoglobulin E Is Likely to Cause Complications of *Mycoplasma pneumoniae* Pneumonia in Children

**DOI:** 10.3389/fcimb.2021.783635

**Published:** 2021-11-24

**Authors:** Lili Zhou, Yuan Li, Zhufei Xu, Xuyun Peng, Xiaoyan Gong, Lin Yang

**Affiliations:** ^1^ Department of Respiratory Medicine, Women and Children’s hospital, Gannan Medical University, Ganzhou, China; ^2^ Department of Respiratory Medicine, The Children’s Hospital, Zhejiang University, Hangzhou, China

**Keywords:** Children, Immunoglobulin E, Mycoplasma pneumoniae, Severe Mycoplasma pneumoniae pneumonia, complications

## Abstract

**Objective:**

To investigate the correlation between serum immunoglobulin E (IgE) levels and the complications in children with *Mycoplasma pneumoniae* pneumonia (MPP).

**Methods:**

A retrospective study of MPP patients hospitalized from May 2019 to July 2021 was performed. We analyzed the clinical manifestations, complications, laboratory findings, and treatments.

**Results:**

A total of 275 patients who met the inclusion criteria were enrolled in the study. We divided patients into two groups based on whether there were complications. Complications occurred in 147 patients, of which pulmonary complications were more common than extrapulmonary complications. The IgE level in the complication group was higher than that in the non-complication group with *p* = 0.041. Patients with complications of necrotizing pneumonitis, pneumothorax, skin rash, or bronchiolitis obliterans had higher IgE levels. There was no statistically significant difference in IgE levels between pulmonary complications and extrapulmonary complications. The older the age, the greater the probability of complications (*p* = 0.001). The group with complications was more likely to have chest pain (*p* = 0.000), while the group without complications was more likely to have wheezing (*p* = 0.017). The use of bronchoscopy and glucocorticoids was higher in the complication group than in the non-complication group (*p* = 0.000).

**Conclusions:**

MPP patients with higher IgE levels had more severe clinical symptoms and complications. We speculated that IgE might be a biomarker for complications after *MP* infection.

## Introduction

*Mycoplasma pneumoniae* (*MP*) is a common pathogen of community-acquired pneumonia in children (Shi et al., 2019). Many literature have reported that the severity of the disease caused by *MP* can range from mild to fatal (Waites and Talkington, 2004; Zhou et al., 2020). *MP* pneumonia (MPP) is mostly self-limited, but it can produce a variety of pulmonary and extrapulmonary complications and can even be life-threatening in some individuals (Poddighe, 2018). Extrapulmonary complications caused by *MP* can occur in any organ in the human body (Fan et al., 2015; Shah et al., 2019). It is uncertain under what circumstances *MP* infection is more likely to cause complications. Recently, in our clinical practice, we found that the total serum immunoglobulin E (IgE) increased in children with *MP* infection. Some studies also found that MPP patients had elevated total serum IgE levels (Poddighe et al., 2018; Ye et al., 2018). However, the correlations between the total serum IgE level and complications of MPP in children are still unclear. Therefore, we decided to analyze the serum IgE level in hospitalized children with MPP in order to confirm these data.

## Patients and Methods

### Study Subjects and Diagnosis

The data of patients with MPP who were admitted to Women and Children’s Hospital of Ganzhou from May 2019 to July 2021 were retrospectively collected. The inclusion criteria were as follows: 1) met the diagnostic criteria: with clinical manifestations (presence of fever, cough, tachypnea, and difficulty in breathing), abnormal lung auscultation, radiologic findings (presence of a new infiltrate on chest radiography or consolidation not attributable to some other etiologies), and *MP* detected to be positive by laboratory tests (Medjo et al., 2014); and 2) age less than 15 years. The exclusion criteria were as follows: 1) children with evidence of coinfection; 2) children with immune deficiency; and 3) children with a history of allergies. Severe MPP (SMPP) was defined as MPP with any one of the following: 1) a poor general condition; 2) fastidium or dehydration; 3) disturbance of consciousness; 4) an increased respiratory rate (infants >70 breaths/min and older children >50 breaths/min); 5) dyspnea; 6) cyanosis; 7) extent of infiltration on chest X-ray ≥2/3 of one lung or multilobe involvement; 8) extrapulmonary complications; 9) pleural effusion; and 10) oxygen saturation in room air ≤92% (Zheng et al., 2021).

### Microbiological Analyses

On the day of admission, 3 ml of venous blood was collected from patients, and the passive agglutination assay was used to detect *MP* antibody (*MP* Antibody Test Kit, Fujitsu Joint Stock Company). It was defined as *MP* positive when the *MP* antibody titer was ≥1:160. Nasopharyngeal aspirates were collected on the day of admission, and *MP*-DNA was detected by PCR (*MP* Nucleic Acid Test Kit, National Sun Yat-sen University). Simultaneous detection of other pathogens was performed through the indirect immunofluorescence assay of respiratory viruses (adenovirus, respiratory syncytial virus, parainfluenza virus 1–3, and influenza virus A and B) using a D3 Ultra DFA Respiratory Virus Screening & ID Kit (Diagnostic Hybrids, Inc., OH, USA) and blood cultures for bacteria (BD FX200 blood culture system).

### Data Collection

By consulting the electronic medical records of all patients, the demographic, clinical, and laboratory data were collected retrospectively. Clinical signs and symptoms of patients, including fever, cough, wheezing, chest pain, and complications, were obtained. All patients underwent chest X-ray or chest CT scan to confirm focal or segmental infiltration, with or without pleural effusion, atelectasis, pneumothorax, pulmonary embolism, and pulmonary necrosis.

### Measurement of Serum Immunoglobulin E

Serum IgE levels were obtained using the automatic biochemical immunoassay analyzer produced by Roche. The test kit was also provided by Roche. The reaction was carried out according to the manufacturer’s instructions.

### Ethical Approval

This study was approved by the Ethics and Research Council of Women and Children’s Hospital of Ganzhou (201905-A01) on May 15, 2019. The data from patients were collected anonymously.

### Statistical Analyses

The statistical analyses were carried out using SPSS 20.0 software package. Continuous variables were reported as the mean ± SD and were compared using Student’s t-test or the non-parametric Mann–Whitney U-test. The categorical variables were shown as number (%) and were compared using the chi-square test. *p*-Values <0.05 were considered to be significant. The figures and graphs were generated by GraphPad Prism 5.

## Results

### The Demographic and Clinical Information

From May 2019 to July 2021, 275 patients admitted to our hospital for MPP were enrolled in the study. The demographic and clinical characteristics are shown in **Table 1**. Of these, 167 (60.7%) were male and 103 (39.3%) female. The median age of the children was 4.93 years, varying from 2 months to 14 years 10 months; 9.4% of the patients were under 12 months, 24% of the patients were between 1 and 3 years, 32.4% of the patients were between 3 and 6 years, and 34.2% of the patients were older than 6 years. A total of 97 patients had SMPP. Almost all the children had cough (99.6%) and fever (95.3%), 24 (8.7%) had wheezing, and 22 (8.0%) had chest pain. Moreover, 98 (35.6%) patients received glucocorticoid therapy, 70 (25.5%) patients received electronic bronchoscopy alveolar lavage, 31 (11.3%) patients required oxygen therapy, 11 (4%) patients received immunoglobulin therapy, and eight (2.9%) patients needed intensive care unit (ICU) treatment. Further, 95 (34.5%) children experienced pulmonary complications, and 52 (18.9%) children experienced extrapulmonary complications.

**Table 1:** Demographic and clinical characteristics of 275 patients with MPP.

Index	N = 275
Demographic characteristics	
Age (years)	4.93 ± 3.12
0–1 year	26 (9.4%)
1–3 years	66 (24.0%)
3–6 years	89 (32.4%)
6–15 years	94 (34.2%)
Sex (male/female)	167/103
Clinical characteristics	
Fever	262 (95.3%)
Cough	264 (99.6%)
Wheezing	24 (8.7%)
Chest pain	22 (8.0%)
SMPP	97 (35.3%)
Pulmonary complications	95 (34.5%)
Extrapulmonary complications	53 (19.3%)
Treatments	
Oxygen therapy	31 (11.3%)
Glucocorticoid	98 (35.3%)
Bronchoscope	70 (25.5%)
Immunoglobulin	11 (4%)
ICU admission	8 (2.9%)

MPP, Mycoplasma pneumoniae pneumonia; SMPP, severe Mycoplasma pneumoniae pneumonia; ICU, intensive care unit.

### Complications and Serum Immunoglobulin E Levels

**Figure 1:**
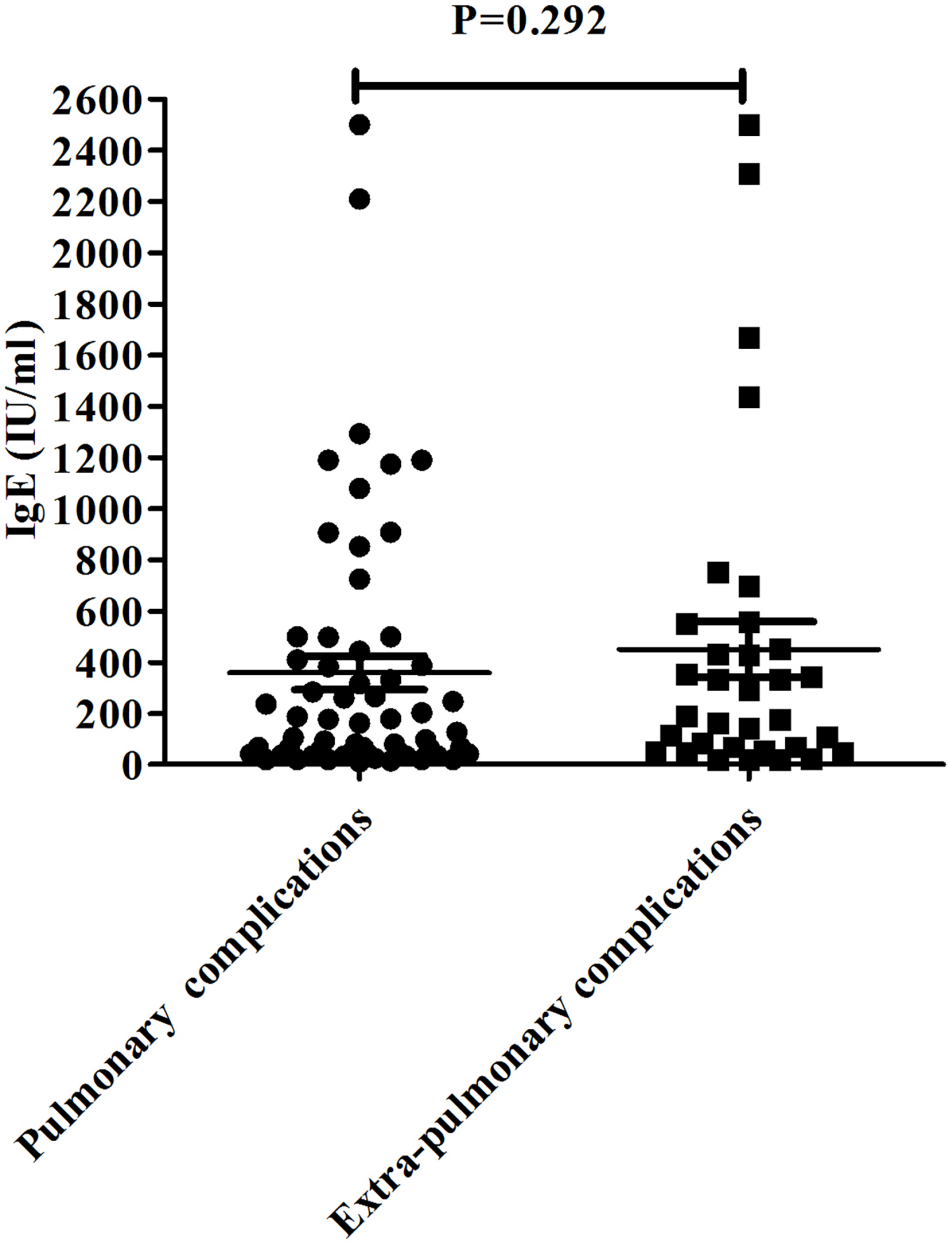
The IgE level in the pulmonary complication group and extrapulmonary complication group.

The pulmonary and extrapulmonary complications are listed in **Table 2**. The IgE levels of patients with different complications varied from 0 to 2,500 IU/ml. Complications occurred in 147 patients, of which pulmonary complications were more common than extrapulmonary complications. Thirty (10.9%) patients had atelectasis, and 55 (20%) had pleural effusion, accounting for 89.5% of pulmonary complications. Extrapulmonary complications were more common in terms of skin rashes and hypokalemia. Further, 21 (7.64%) patients had hypokalemia, 12 (4.4%) patients had skin rashes, six (2.18%) patients had myocarditis, and six (2.18%) patients had hepatitis. Patients with complications of necrotizing pneumonitis, pneumothorax, skin rashes, eye pain, or bronchiolitis obliterans had higher IgE levels. No statistically significant difference was found in IgE levels between pulmonary complications and extrapulmonary complications with *p* = 0.292, as shown in **Figure 1**.

**Table 2:** IgE levels of patients with different complications.

	Case (n, %)	IgE level (IU/ml)
Pulmonary complications		
Atelectasis	30 (10.9%)	361.65 (0–2,211)
Pleural effusion	55 (20%)	237.36 (0–1,190)
Necrotizing pneumonitis	3 (1.1%)	1,689 (67–2,500)
Pneumothorax	1 (0.36%)	2,500
Bronchiolitis obliterans	2 (0.73%)	764.95 (236–1,294)
Plastic bronchitis	4 (1.5%)	254.66 (11–498)
Pulmonary embolism	0	–
Extrapulmonary complications		
Skin rash	12 (4.4%)	782.71 (46–2,500)
Myocarditis	6 (2.18%)	59.8 (23–109)
Hepatitis	6 (2.18%)	18.55 (11–23)
Reactive arthritis	3 (1.09%)	140 (110–167)
Encephalitis	4 (1.5%)	187.35 (69–452)
Hypokalemia	21 (7.64%)	453.19 (19–2,310)

### Comparisons Between the Groups With Complications and Without Complications

**Figure 2:**
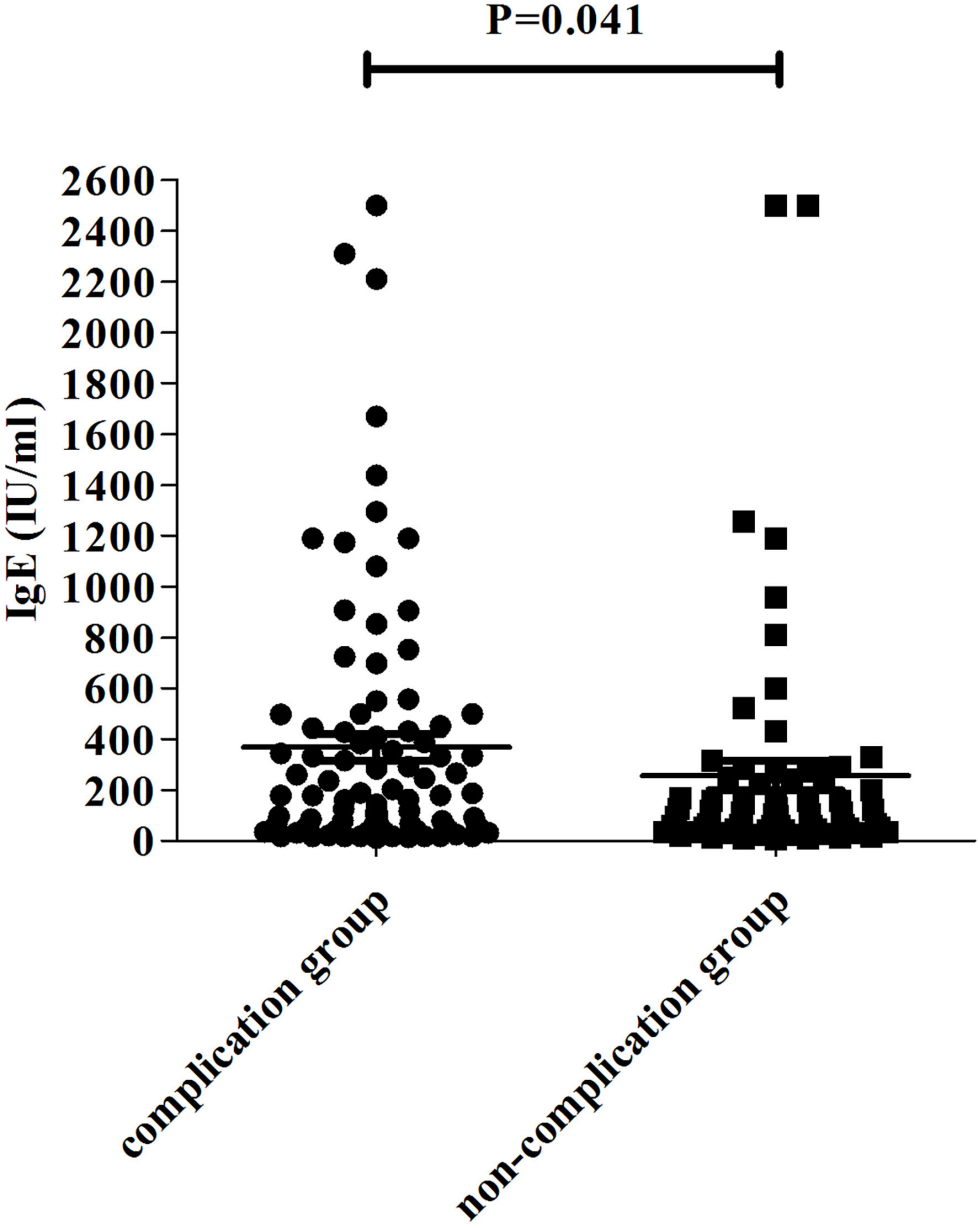
The IgE level in the complication group and the non-complication group.

**Figure 3:**
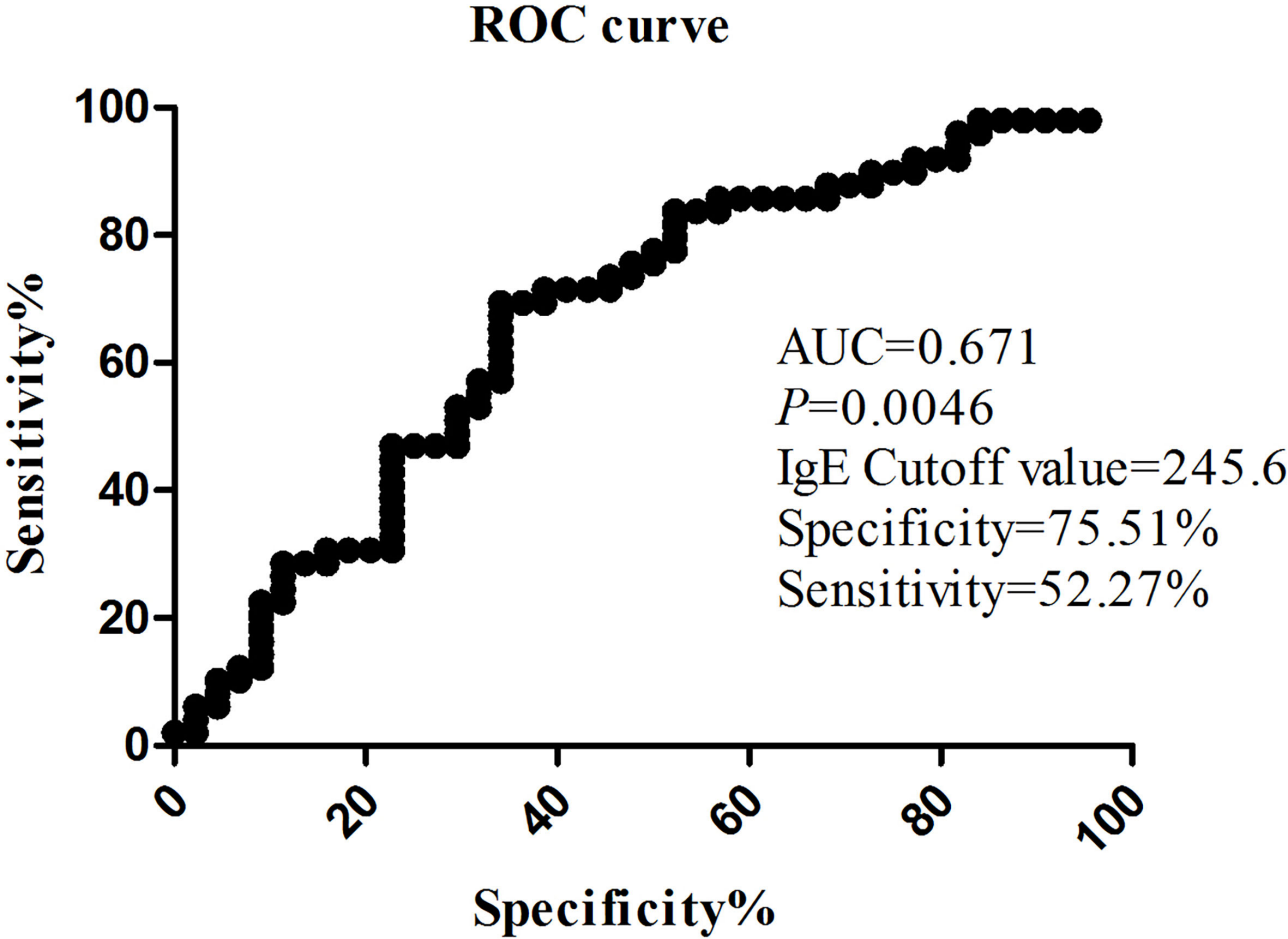
The value on the receiver operating characteristic (ROC) curve of the IgE level between the complication group and the non-complication group.

Patients were divided into two groups according to the presence or absence of complications. The demographics, clinical characteristics, laboratory findings, and treatments of children in the complication and non-complication groups are shown in **Table 3**. The children in the complication group were much older than those in the non-complication group (*p* = 0.001). No statistically significant difference in the appearance of fever and cough symptoms was observed between the two groups. Higher incidence of chest pain (*p* = 0.000) and lower incidence of wheezing (*p* = 0.017) were found in the complication group than in the non-complication group. Significant differences in fever duration, hospitalization duration, and incidences of SMPP between the two groups (*p* < 0.001) were also found. The probability of SMPP in patients with complications was higher than that in patients without complications (*p* = 0.000). As shown in **Figure 2**, the IgE level fluctuated greatly from patient to patient. The IgE level in the complication group was higher than that in the non-complication group (*p* = 0.041). The risk factors for complications by multifactor binary logistic regression analysis were analyzed. After age factors were adjusted, a significant difference in the IgE level was noted between the complication and non-complication groups (*p* = 0.047), as shown in **Table 4**. The threshold of IgE between these two groups was determined using the receiver operating characteristic (ROC) curve. The area under the ROC curve was 0.671. When the cutoff value for the IgE level was set at 245.6 IU/ml, the specificity and sensitivity were 75.51% and 52.27%, respectively (**Figure 3**). Besides the IgE level, D-dimer, lactate dehydrogenase (LDH), and ferritin levels also differed significantly between the two groups (*p* < 0.01). However, no significant difference was observed in white blood cell count, neutrophil percentage, hemoglobin, platelets, erythrocyte sedimentation rate, and the levels of immunoglobulin A, immunoglobulin G (IgG), immunoglobulin M, alanine transaminase (ALT), aspartate transaminase (AST), and creatine kinase isoenzymes (CK-MB). Patients in the complication group were more likely to receive oxygen therapy, glucocorticoids, and bronchoscopy as compared with those in the non-complication group (*p* < 0.01). Meanwhile, eight patients required ICU treatment, and one required mechanical ventilation. Except for one child who died, the rest of the children recovered and were discharged from the hospital.

**Table 3:** The demographics, clinical characteristics, laboratory findings, and treatments of children in the complication group and the non-complication group.

	Complication group (n = 147)	Non-complication group (n = 128)	*p*
Sex (male)	88 (59.9%)	79 (61.7%)	0.805
Age (years)	5.54 ± 3.12	4.23 ± 2.93	0.001
Clinical presentation			
Fever	142 (96.6%)	120 (93.8%)	0.394
Cough	147 (100%)	127 (99.2%)	0.462
Wheezing	7 (4.8%)	17 (13.3%)	0.017
Chest pain	21 (14.3%)	1 (0.8%)	0.000
Length of fever (days)	7.49 ± 4.11	5.68 ± 3.7	0.000
Length of stay (days)	8.05 ± 3.3	6.34 ± 1.38	0.000
SMPP	79 (53.7%)	18 (14.1%)	0.000
Laboratory findings			
IgE level (IU/ml)	359.96 ± 544.26	213.55 ± 380.36	0.041
D-dimer (mg/L)	2.34 ± 4.37	1.01 ± 1.28	0.005
White blood cell (×10^9^/L)	9.00 ± 4.00	9.83 ± 4.45	0.11
Neutrophil (%)	60.13 ± 18.19	56.81 ± 17.97	0.13
Hb (g/L)	121.67 ± 9.74	120.24 ± 10.14	0.24
PLT (×10^9^/L)	317.55 ± 97.75	324.67 ± 109.31	0.48
CRP (mg/L)	27.35 ± 94.57	12.53 ± 16.08	0.065
LDH (U/L)	335.04 ± 110.18	294.67 ± 63.5	0.005
Ferritin (ng/ml)	297.34 ± 289.77	97.12 ± 39.09	0.001
ESR (mm/h)	28.18 ± 19.3	25.21 ± 17.55	0.34
AST (U/L)	24.81 ± 21.08	26.47 ± 20.0	0.51
ALT (U/L)	45.12 ± 32.81	49.48 ± 33.88	0.29
IgA (g/L)	2.23 ± 1.91	2.07 ± 1.74	0.31
IgG (g/L)	7.79 ± 3.24	8.6 ± 3.51	0.098
IgM (g/L)	2.13 ± 7.98	2.94 ± 12.3	0.62
CK-MB (U/L)	25.87 ± 17.07	22.76 ± 14.98	0.11
Treatments			
Oxygen therapy	24 (16.3%)	7 (5.5%)	0.007
Glucocorticoid	74 (50.3%)	24 (18.8%)	0.000
Bronchoscope	57 (38.8%)	13 (10.2%)	0.000
Immunoglobulin	8 (5.4%)	3 (2.3%)	0.23
ICU admission	7 (4.8%)	1 (0.8%)	0.072

SMPP, severe Mycoplasma pneumoniae pneumonia; Hb, hemoglobin; PLT, platelets; CRP, C-reactive protein; LDH, lactate dehydrogenase; ESR, erythrocyte sedimentation rate; AST, aspartate transaminase; ALT, alanine transaminase; CK-MB, creatine kinase isoenzymes; ICU, intensive care unit.

**Table 4:** Logistic regression analysis of complications in children with MPP.

	β	SE	*p*	OR	95% CI
Age	0.197	0.057	0.001	11.846	1.089–1.363
IgE	0.001	0.000	0.047	3.626	1.000–1.002

MPP, Mycoplasma pneumoniae pneumonia.

### Analysis of 14 Cases With Particularly High Immunoglobulin E

There were some patients with abnormally increased IgE (cases with IgE above 1,000 IU/ml) in our study. The information of these 14 patients is listed in **Table 5**. These patients were all acutely infected, mainly manifesting with fever and cough. There were four children younger than 3 years: two of them had wheezing, and one had complication of bronchiolitis obliterans. Two of the 14 children with elevated IgE had no complication. It was found that the older the patients, the more likely to have complications. Pulmonary complications were more common in patients with high IgE, and prognosis was good after active treatment. But unfortunately, of these 14 patients, one died due to multiple complications.

**Table 5:** Information of 14 patients with particularly high IgE.

Case	Sex	Age (years)	Personal history and allergies	Chief complaint on admission	Physical examination	IgE (IU/ml)	MP antibody titer	Complications	Main treatment	Outcome
1	Male	6	Three times respiratory tract infection and no allergies	Cough for 1 week and fever for 5 days	Shortness of breath and left lung breath sounds weakened	1,190	1:320	Pleural effusion and left lung atelectasis	AZM, CAZ, MP	Discharged
2	Female	12.9	Five times respiratory tract infection and no allergies	Fever for 10 days and cough for 9 days	Left lung breath sounds weakened and no rales	1,257	1:640	Hypokalemia and left atelectasis	AZM	Discharged
3	Male	8.8	Diarrhea, hand, foot and mouth disease, and no allergies	Fever and cough for 8 days	Left lung breath sounds weakened and no rales	1,670	1:320	Rash	E, PAT	Discharged
4	Male	2.5	None	Fever for 9 days and cough for 7 days	Right lung breath sounds weakened and no rales	1,257	1:320	None	E, PR	Discharged
5	Male	2	Pneumonia and no allergies	Fever and cough for 4 days	Shortness of breath, three-concave sign, white discharge in the tonsils, and no rales	2,211	1:640	None	AZM, MP, Oxygen	Discharged
6	Female	11.3	Encephalitis, falls and fractures, and no allergies	Fever and cough for 8 days	Right lung breath sounds weakened and no rales	1,080	1:640	Atelectasis	AZM, BAL	Discharged
7	Female	3.1	Diarrhea and no allergies	Cough for 15 days and fever for 12 days	Left lung breath sounds weakened and no rales	1,437	1;320	None	AZM	Discharged
8	Female	8.8	Five to six times respiratory tract infection and no allergies	Fever and cough for 2 weeks	Right lung breath sounds weakened and no rales	1,190	1:320	Rash	AZM, MP	Discharged
9	Male	6.4	None	Fever for 10 days and cough for 8 days	Right lung breath sounds weakened and wet rales	2,500	1:1,280	Right pleural effusion, right atelectasis, respiratory failure, right tension pneumothorax, necrotizing pneumonia, and thrombosis	AZM, IPM, MP, thoracentesis, closed thoracic drainage, BAL	PICU Death
10	Female	13	Appendicitis and no allergies	Fever and cough for 2 weeks	Right lung breath sounds weakened, no rales	1,190	1:640	Pleural effusion	AZM, CAZ, MP	Discharged
11	Male	5	Kawasaki disease, three times respiratory tract infection, and no allergies	Fever for 4 days, cough for 3 days, and wheezing for 1 days	Wheezing in both lungs, prolonged expiratory phase	2,310	1:320	Hypokalemia	AZM, PR	Discharged
12	Female	8.7	Hemangioma, respiratory tract infection once a year and no allergies	Cough for 10 days	Left lung breath sounds weakened	1,174	1:320	Left pleural effusion	AZM	Discharged
13	Male	1.25	Congenital laryngomalacia and no allergies	Cough and fever for 4 days and shortness of breath for 2 days	Shortness of breath, wet rales and wheezing in both lungs	1,294	1:160	Bronchiolitis obliterans	AZM, CAZ, PAT, BAL	Recurrent wheezing
14	Female	1.4	Bronchopulmonary dysplasia and no allergies	Wheezing for 10 days and cough for 8 days	Shortness of breath, wheezing in both lungs, wet rales in the right lung	2,500	1:320	Rash	AZM, CAZ, BAL	Discharged

AZM, azithromycin; CAZ, ceftazidime; MP, methylprednisolone; E, erythromycin; PAT, prednisone acetate tablets; PR, Pulmicort Respules; IPM, imipenem; BAL, bronchoscopy and alveolar lavage.

## Discussion

The frequency of pulmonary and extrapulmonary symptoms associated with *MP* infection has increased in recent decades. Few studies reported the incidence of various complications caused by *MP*. Most studies were in the form of case reports or reviews. The incidence of pulmonary complications in this study was 34.5%, which was slightly higher than that reported in the literature. Xu reported that the incidence of pulmonary complications in their study was 20.65% (Xu et al., 2021). The extrapulmonary complication rate in this study was 18.9%, which was a little lower than reported. Previous studies found that the incidence of extrapulmonary injury during *MP* infection could reach 25%–50% (Gong et al., 2021). Zheng reported that the incidence of extrapulmonary complication was 33.4% (Zheng et al., 2021). The most common extrapulmonary complication in our study was hypokalemia. However, few people pay attention to hypokalemia caused by *MP*. We found that 7.64% (21 in 275) patients had hypokalemia. Han reported that 15.7% (77 in 489) patients had hypokalemia, which was associated with SMPP, fever duration, and hospitalization duration (Han et al., 2021). We found that 4.4% (12 in 275) patients had skin rashes. *MP* infection could easily cause skin rashes, as described in previous reports (Waites and Talkington, 2004; Narita, 2010). And three patients suffered from reactive arthritis in this study, which indicated that *MP* could induce arthritis in some people. This was in contrast to previous reports (Shokrollahi et al., 2017; Mărginean et al., 2021). Another study showed that increased total serum IgE was associated with reactive arthritis in patients with *MP* infection (Poddighe et al., 2021). Encephalitis, which affected children, was the most frequent neurological complication of *MP* (Arighi et al., 2018). Four children suffered from encephalitis in this study. Qing reported that 18.6% (11 in 59) patients had abnormal ALT concentrations (>40 U/L) and that 55.9% (33 in 59) patients had abnormal CK-MB concentrations (>28 U/L) (Fan et al., 2015). In this study, 2.18% (six in 275) patients had myocarditis, and 2.18% (six in 275) patients had hepatitis. The incidence of myocarditis and hepatitis was much lower than previously reported, maybe because we defined abnormal elevation of ALT and CK-MB to be three times higher than normal. Also, our sample size was larger than that of previous reports.

Few studies reported both pulmonary and extrapulmonary complications, and even fewer reported on the role of IgE levels in the occurrence of complications in MPP. We found no statistically significant difference in IgE levels between pulmonary and extrapulmonary complications. However, Poddighe reported that the serum IgE level of hospitalized children with *MP*-related extrapulmonary diseases was significantly higher than that of children with only respiratory diseases (Poddighe et al., 2018). In this study, the serum IgE levels were relatively high in patients with MPP having complications. As far as we know, the current studies on IgE were all related to allergies, and few of them focused on the significance of elevated IgE in patients with MPP having complications. *MP* induced allergy by producing P1-specific IgE; therefore, *MP* was not only a source of infection but also an allergen for some people (Ye et al., 2018). The persistent *MP* antigen stimulation and/or invasion greatly increased the possibilities of severe lung lesions and pulmonary and extrapulmonary complications (Zhou et al., 2014). As an antigen, *MP* can produce specific antibodies in the body; it has common antigens with the body’s heart, lungs, brain, and so forth. It can produce auto-antibodies. The antigen and antibodies form complexes and then activate complement and immune cells to exert powerful immune and autoimmune responses (Waites and Talkington, 2004). It was speculated that the tissue damage and systemic inflammation in children with MPP having an increased IgE level were more severe, and therefore, the fever course was longer, and pulmonary and extrapulmonary complications were more common.

So far, the pathogenesis of *MP* was still not clear. It was mainly related to the theory of immunological pathogenesis, adsorption of respiratory epithelial cells, and direct invasion of *MP* (Meyer et al., 2014). The current theory that excessive immune response led to the progress of MPP was generally accepted (Yan et al., 2019). The humoral immunity and cellular immune dysfunction played an important role in the pathogenesis of *MP*, which could cause serious complications of multiple systems outside the lungs and could prolong the course of the disease (Shi et al., 2019). Investigations on IgE production are important to understand the cellular mechanisms leading to increased IgE levels in patients with MPP (Ye et al., 2014). Abnormally elevated IgE levels were considered to be one of the markers of immune imbalance (Magen et al., 2014; Poddighe and Marseglia, 2016). Thus, we speculated that IgE might be a biomarker for complications after *MP* infection (Gu et al., 2020).

This study had several limitations. First, this was a retrospective study with some inevitable selection biases. Second, the sample size was limited, with single-center data. Third, large-sample prospective studies needed to be designed to verify the reliability of this retrospective study in the future. In summary, patients with MPP having higher IgE levels had more severe clinical symptoms and complications. We speculated that IgE might be a biomarker for complications after *MP* infection. This study provided a reliable theoretical basis for early identification and intervention of MPP with elevated IgE levels. Paying attention to the increase in the total serum IgE level in children with MPP can reduce the occurrence of various pulmonary and extrapulmonary complications.

## Data Availability Statement

The original contributions presented in the study are included in the article/supplementary material. Further inquiries can be directed to the corresponding author.

## Ethics Statement

The studies involving human participants were reviewed and approved by Ethics and Research Council of Women and Children’s Hospital of Ganzhou (201905-A01). Written informed consent to participate in this study was provided by the participants’ legal guardian/next of kin. Written informed consent was obtained from the individuals, and minors’ legal guardian/next of kin, for the publication of any potentially identifiable images or data included in this article.

## Author Contributions

LZ contributed to the concept and assisted in the critical writing. YL contributed to design of the study. ZX and XP helped in analyzing and interpreting the data. XG and LY contributed to the collection of clinical information. All authors contributed to the article and approved the submitted version.

## Funding

The project was supported by grants from Bethune Foundation (SCE041BS).

## Conflict of Interest

The authors declare that the research was conducted in the absence of any commercial or financial relationships that could be construed as a potential conflict of interest.

## Publisher’s Note

All claims expressed in this article are solely those of the authors and do not necessarily represent those of their affiliated organizations, or those of the publisher, the editors and the reviewers. Any product that may be evaluated in this article, or claim that may be made by its manufacturer, is not guaranteed or endorsed by the publisher.

## Abbreviations

*MP*, *Mycoplasma pneumoniae*; MPP, *Mycoplasma pneumoniae* pneumonia; SMPP, severe *Mycoplasma pneumoniae* pneumonia; IgE, immunoglobulin E; ICU, intensive care unit; Hb, hemoglobin; PLT, platelets; ESR, erythrocyte sedimentation rate; IgA, immunoglobulin A; IgG, immunoglobulin G; IgM, immunoglobulin M; ALT, alanine transaminase; AST, aspartate transaminase; CK-MB, creatine kinase isoenzymes; ROC, receiver operating characteristic.
